# Explaining socioeconomic inequality in cervical cancer screening uptake in Malawi

**DOI:** 10.1186/s12889-022-13750-4

**Published:** 2022-07-18

**Authors:** Gowokani Chijere Chirwa

**Affiliations:** 1 Economics Department, University of Malawi, Zomba, Malawi; 2 Health Economics and Policy Unit (HEPU), Kamuzu University of Health Sciences, Blantyre, Malawi

**Keywords:** Cervical cancer, Erreygers index, Inequality, Concentration index, Malawi

## Abstract

**Background:**

Cervical cancer is a prevalent public health concern and is among the leading causes of death among women globally. Malawi has the second highest cervical cancer prevalence and burden in the world. Due to the cervical cancer burden, the Malawi government scaled up national cancer screening services in 2011, which are free for all women. This paper is the first study to examine the socioeconomic inequality in cervical cancer screening uptake using concentration indices, in Malawi. Furthermore, it decomposes the concentration index to examine how each factor contributes to the level of inequality in the uptake of cervical cancer screening.

**Methods:**

The data used in this paper were obtained from the nationally representative Malawi Population HIV Impact Assessment (MPHIA) household survey, which was conducted in 2015. Concentration curves were constructed to explore whether there was any socioeconomic inequality in cervical cancer screening and, if so, its extent. This was complemented by concentration indices that were computed to quantify the magnitude of socioeconomic inequality. A decomposition analysis was then conducted to examine the factors that explained/were associated with greater socioeconomic inequality in cervical cancer screening. The methodology in this paper followed that of previous studies found in the literature and used the wealth index to measure socioeconomic status.

**Results:**

The results showed that the concentration curves lay above the line of equality, implying a pro-rich inequality in cervical cancer screening services. Confirming the results from the concentration curves, the overall concentration index was positive and significant (0.142; %95 CI = 0.127, 0.156; *p* < 0.01). The magnitude was lower in rural areas (0.075; %95CI = 0.059, 0.090; *p* < 0.01) than in urban areas (0.195; %95CI = 0.162, 0.228 *p* < 0.001). After undertaking a decomposition of the concentration index, we found that age, education, rural or urban location, and wealth status account for more than 95% of the socioeconomic inequality in cervical cancer uptake.

**Conclusion:**

Despite the national scale-up of free cancer care at the point of use, cervical cancer screening uptake in Malawi remains pro rich. There is a need to implement parallel demand-side approaches to encourage uptake among poorer groups. These may include self-testing and mobile screening centres, among others.

**Supplementary Information:**

The online version contains supplementary material available at 10.1186/s12889-022-13750-4.

## Background

There has been a recent surge of noncommunicable diseases (NCDs) in many low- and middle-income countries (LMICs), which has led to a huge economic burden on households [1]. It is estimated that by 2030, NCDs will account for almost 75% of all deaths globally. Of these deaths, it is estimated that 80% will be in LMICs. Among the many NCDs, cervical cancer has been increasing in LMICs, accounting for 85% of all cases and cancer deaths [2, 3]. Approximately 90% of deaths from cervical cancer occurred in LMICs [4]. Cervical cancer is caused by a human papillomavirus (HPV) infection [5], whereby the cervix cells uncontrollably grow and form tumours arising from abnormality [6].

Cervical cancer has become a modern health concern such that cervical cancer screening and prevention has been set to become one of the last frontiers of universal health coverage (UHC) [7, 8]. UHC is one of the sustainable development goals, which aims to ensure that people can access the services they need while having some form of financial protection. Worldwide, the burden of cervical cancer is the fourth most frequently occurring cancer in women. In 2018, there were an estimated 570,000 new cases, representing 7.5% of all female cancer deaths that were caused by cervical cancer. Additionally, there are more than 311,000 deaths from cervical cancer every year and more than 85% of these occur in less developed regions [9]. In this case, it means that cervical cancer may potentially be an obstacle to the achievement of the SDG on access to healthcare for all due to the financial burden and economic costs associated with it.

The total economic loss due to women’s cancer is estimated to be 4% of the global Gross Domestic Product [10]. By the year 2010, it has been estimated that the cost to the global economy of cervical cancer is around USD 2.7 billion, and this is projected to rise to USD 4.7 billion by 2030 [11]. Furthermore, between 1990 and 2017, the global estimates for years lived with disability (YLDs) (thousand) of cervical cancer was estimated to be 288·1 (confidence interval: 205·6 to 381·5) [12]. From these few examples, it can be said that it is necessary that governments understand the socioeconomic aspects of cervical cancer and should therefore invest more in addressing the global burden of cervical cancer. Doing so would reduce the profound impact of cervical cancer on premature death and disability, as well as substantial and long-lasting social, financial and economic consequences for the affected women, their immediate families and the wider community.

Cervical cancer is preventable [13], and the WHO aims to reduce the annual age-standardised incidence rate of cervical cancer to less than 4 cases per 100,000 women worldwide. To achieve this, the WHO has employed a strategy of vaccinating 90% of all girls by age 15 years, screening 70% of women twice in the age range of 35–45 years, and treating at least 90% of all precancerous lesions detected during screening [14]. One way to achieve the curtailment of cervical cancer screening is screening at proper medical facilities [15]. In response to cervical cancer, it is recommended that females aged 9–14 years receive HPV vaccination before becoming sexually active [16].

Malawi provides a compelling case for the analysis of socioeconomic inequalities in cervical cancer uptake for several reasons. Firstly, cancer cases are increasing in Malawi [17]. Secondly, Malawi has the second highest cervical cancer incidence globally, with prevalence among the female population at 75.9 cases per 100,000 [18], which has resulted in higher cancer-related mortality cases. Thirdly, in 2004 the Malawi government started cervical cancer screening as a pilot project in Mulanje district. Given the success of the pilot project, massive health reform was undertaken in 2011, where screening services were scaled up to central hospitals and district hospitals. Non-governmental hospitals also adopted the intervention [19]. Evidence indicates that following the expansion of services between 2011 and 2015, the number of cervical cancer screening sites increased from 75 to 130 per year, and the number of women screened grew from 15,331 to 49,301. Furthermore, almost 145,015 women were screened [19]. Fourthly, after 2011, media campaigns were launched to raise public awareness of the availability of cervical cancer screening services [20].

Studies on socioeconomic inequality in cervical cancer screening are scarce in low-income countries. On another front, a different study identified factors that hinder uptake of cervical cancer screening in most sub-Saharan African countries. The factors identified include lack of knowledge about cervical cancer, gaps in education and training among providers and lack of resources and health infrastructure at the facility level and within the overall health system [21]. In Kenya, among various factors, it was observed that gender equity and health insurance were among the huge contributors to the wealth-related inequality in cervical cancer screening [22]. Though important, the health insurance aspect may be immaterial in Malawi, given that only about 3 % of the population owns health insurance and is private, since no public insurance scheme exists in Malawi. The Kenyan findings corroborate what was found in individual countries such as Ethiopia, Burkina Faso, Gabon and Sudan, i.e., that a low level of knowledge concerning cervical cancer, and availability of screening services, personal or psychological convictions, cost associated with screening and treatment as well as a low level of income/wealth were the individual-level barriers [23–29]. In Mozambique, a low prevalence in cancer screening was observed, and the number of schooling years and use of oral contraceptives were factors associated with uptake [30]. In Uganda, uptake was said to be associated with receipt of HIV care at a level II health centre, knowledge of cervical screening and low perception of risk of cervical cancer [31]. Coupled with the above, other barriers to uptake emanate from the supply side, for example, the lack of adequate testing facilities, health professionals and funding, to mention only a few [32–34].

One of the earlier studies on socioeconomic inequality in cervical cancer screening, which used data from the 2002 World Health Survey, suggests that cervical cancer screening was concentrated among wealthier women [35] in the 67 countries. However, Malawi was not included in the analysis of this study. Apparently, 2002 is long before cervical cancer screening was piloted in Malawi and the first data on cervical cancer screening uptake was collected. A paper closely related to the current study [36] established that wealth-related inequalities of women’s knowledge of cervical cancer screening and service utilisation in 18 resource-constrained countries is concentrated on the wealthier. As was the case in the other study, this paper did not include Malawi in its analysis because Malawi data never included screening for cervical cancer at that point of the available data. The literature on Malawi is scarce, given that issues of cancer screening have only been included very recently in national programmes. Other studies have taken an ethnographic approach in understanding the behavioural, cultural and societal influences regarding cervical cancer. Findings suggest that people seek cancer screening only when symptoms persist or when they become ill. Furthermore, there is also low awareness of cervical cancer and screening services among the service providers as well as the population [17, 20, 37].

In light of the above arguments, this paper contributes to the current literature on cervical cancer screening by assessing socioeconomic inequalities in cervical cancer screening in Malawi. This is in view of the recent scale-up of cancer screening services and the incorporation of cancer screening services as part of the new public health intervention [38]. This paper starts by assessing the factors affecting the uptake of cervical cancer in Malawi. Further, to deepen our understanding and to highlight areas for policy action, the paper assesses factors that significantly explain socioeconomic inequality (by decomposing the socioeconomic inequality index) using data from the Malawi HIV and AIDS Impact Assessment 2015–16 (MPHIA). The results show that cervical cancer screening is highly concentrated among the rich. Furthermore, socioeconomic inequality in cervical cancer screening uptake is higher in urban areas than it is in rural areas.

## Materials and methods

### Data

The study used secondary data from the Malawi Population-Based HIV Impact Assessment (MPHIA). This was a household-based national survey conducted between November 2015 and August 2016. The survey project aimed to measure the status of Malawi’s national HIV response. This was the first survey in Malawi which measured national HIV incidence, paediatric HIV prevalence and viral load suppression. In terms of design, the MPHIA used a two-stage stratified cluster approach based on the 2008 Malawi Population and Housing Census. The first stage involved the selection of 500 EAs (clusters) using a probability proportional to size method. In the second stage, a sample of households was randomly selected within each EA, or cluster.

Among others, the MPHIA offered HIV counselling and testing with a return of results and collected information about uptake of care and treatment services. MPHIA was a collaborative project led by the Malawi government through the Ministry of Health (MOH), conducted with funding from the U.S. President’s Emergency Plan for AIDS Relief (PEPFAR) and technical assistance through the U.S. Centers for Disease Control and Prevention (CDC). The survey was implemented by ICAP at Columbia University in collaboration with local partners, including the Centre for Social Research (CSR) at the University of Malawi, the National Statistical Office (NSO) and the College of Medicine—Johns Hopkins Project (COM-JHP) at the University of Malawi. The datasets analysed during the current study are publicly available at MPHIA, https://phia-data.icap.columbia.edu/datasets?country_id=3, and free to download.

### Ethical clearance

The survey was conducted in accordance with the World Medical Association Declaration of Helsinki [39]. Ethical clearance for the study was gained by the Institution Review Boards from Malawi and USA. These include the National Health Sciences Research Committee in Malawi (NHSRC) and the Institutional Review Boards at the Centers for Disease Control and Prevention (CDC; Atlanta, USA), Columbia University Medical Center and Westat (a statistical survey research organisation). Respondents’ consent was obtained using an electronic tablet, where the participant had to sign or use a fingerprint as a sign of agreement. The respondents were recruited only when they consented to participate in the study. During the consent process, respondents were told in advance that the data would be used in future research. Data were anonymised in order to allow for secondary data use.

### Dependent variable

The variable of interest in the paper is cervical cancer screening uptake. Respondents were asked if they ever had been examined by a healthcare provider to check for cervical cancer. The tests respondents were asked about were pap smear, HPV test and VIA test. As a reminder, survey respondents were told that for a pap smear and HPV test, a healthcare provider puts a small stick inside the vagina to wipe the cervix and sends the sample to the laboratory. For a VIA test, a healthcare worker puts vinegar on the cervix and looks to see if the cervix changes colour. If a respondent indicated that they had undergone a cervical cancer test, the variable was coded as 1; otherwise, the variable was coded as 0.

### Independent variables

The paper follows the aforementioned literature from Malawi and other similar countries to select the covariates that have been shown to be associated with cervical cancer uptake and knowledge [19, 40–43]. These include a place of residence, age, region, education, religion, work status and whether the respondent was ever tested for HIV. The variable work status was used to capture whether an individual is employed or not. If working, a value of 1 was assigned and if not, 0. An individual’s location of residence was captured as rural or urban. If urban, a value of 1 was assigned and if rural, 0. All married individuals were assigned a value of 1 and if unmarried, 0. The education status level of individuals was categorical and classified as none (=1, if no education and 0 otherwise), primary (=1, if primary and 0 otherwise), secondary (=1, if secondary and 0 otherwise) or post-secondary (=1, if post-secondary and 0 otherwise). To control for religion, individuals were classified as Christians (=1, if Christian and 0 otherwise), Muslim (=1, if Muslim and 0 otherwise) or no religion (=1, if no religion and 0 otherwise). Taking into account the role of HIV programmes in Malawi, which have been integrated with screening for cervical cancer, individuals were also asked if they ever tested for HIV. If an individual indicated that they had been tested for HIV, a value of 1 was assigned and 0 otherwise. Region was included to capture spatial issues; thus, three regions were used: North (=1, if North and 0 otherwise), Central (=1, if Central and 0 otherwise) and South (=1, if South and 0 otherwise).

### Socioeconomic status

In order to capture household socioeconomic status, the paper used the wealth index [44]. The wealth index is the conventional measure used if there is no household expenditure and income data [44–46]. Following the conventional approach, the MPHIA study collected data on durable assets in line with demographic health surveys. The index itself was constructed using principal component analysis [44, 46]. The advantage of this is that the index can be used for international comparison [47]. The index was then categorised into five quintiles: poorest (wealth quintile 1); poorer (wealth quintile 2); middle (wealth quintile 3); rich (wealth quintile 4); richer (wealth quintile 5). The variables used in the construction of the wealth index are reported in Additional file 1: Appendix 1.

### Statistical analysis

Firstly, in order to assess the determinants of cervical cancer uptake, the study used a logit regression model following the econometrics literature [48–51]. Cervical cancer uptake can take two values, not undertaking cervical cancer screening (*Y*_*i*_ =0) or undertaking cervical cancer screening (*Y*_*i*_ =1). Therefore, let *P*_*i*_ represent the probability of an individual undertaking cervical cancer screening, then the probability of not undertaking cervical cancer screening is given as 1 − *P*_*i*_. Since we do not observe *P*_*i*_ but the outcome *Y*_*i*_ =1 if an individual has undertaken cervical cancer screening and *Y*_*i*_ =0 if not, then the following probabilities apply:

1 $$\mathit{\Pr}\left({Y}_i=1\right)={P}_i$$

2 $$\mathit{\Pr}\left({Y}_i=0\right)=1-{P}_i$$

The probability of an individual undertaking cervical cancer screening is given as

3 $${P}_i=E\left({Y}_i=1|X\right)=\frac{1}{1+{e}^{-\left({\beta}_0+{\beta}_k{x}_{ik}\right)}}$$

The probability of an individual not undertaking cervical cancer screening is thus given as

4 $$\left(1-{P}_i\right)=E\left({Y}_i=0|X\right)=\frac{1}{1+{e}^{\left({\beta}_0+{\beta}_k{x}_{ik}\right)}}$$

The marginal effects from eqs. (4) are calculated using Stata’s inbuilt *margins* command. For a technical derivation of marginal effects and discussion, see Wooldridge (2010).

Secondly, to measure and explain socioeconomic inequality in cervical cancer screening, this paper used the concentration index (CI) and decomposition method [45]. The CI has been used to measure socioeconomic inequality in various health contexts [52–57]. The CI is estimated as follows:

5 $$\mathrm{CI}=\frac{2}{\mu}\mathit{\operatorname{cov}}\left({y}_i,{r}_i\ \right)$$

where *y*_*i*_ is indicator of cervical cancer testing *i*, *r*_*i*_ is the fractional ranking of individuals according to the wealth index and *μ* is the mean of *y*_*i*_. The CI value ranges between − 1 and 1. If the CI is positive, it indicates a greater utilisation of cervical cancer screening among the rich (pro-rich). If it is negative, it indicates higher utilisation of cervical cancer testing among the poor (pro-poor). The higher the absolute value of the CI, the greater the extent of inequality. The range of the CI decreases when the variable of interest is a binary indicator. This is because of the lower and upper bounds of the CI depending on the mean of the outcome variable [58]. Therefore, the change in socioeconomic inequality measured by the CI can be affected considerably if the mean of the variable of interest changes over time [59]. Wagstaff [58] proposed the following correction of the CI, known as Wagstaff’s Index (WI), to address this problem:

6 $$WI=\frac{CI}{1-\mu }$$

The WI is an ad hoc way to normalise the CI, and it could “blow up the levels of measured inequality for distributions with either high or low means” (Erreygers, 2009b, p. 523) [59]. Therefore, Erreygers (2009a) suggested alternative normalisation of the CI, referred to as the Erreygers Index (EI):

7 $$EI=4\mu CI$$

The interpretation of both the WI and the EI is like the standard CI. To understand the drivers of socioeconomic inequality, we also employed the decomposition technique to partition socioeconomic inequality in healthcare into the socioeconomic inequalities in the determinants of healthcare. Assuming *y*_*i*_, utilisation of delivery care services is modelled by an additively separable linear function of *X*_*j*_ (a vector of covariates) as shown below:

8 $${y}_i=\alpha +\sum \limits_{j=1}^J{\beta}_j{X}_{ji}+{\varepsilon}_i$$

As shown by Wagstaff et al. (2003) [60], the CI in eq. (7) can be decomposed into the weighted sum of the socioeconomic inequality in the determinants for cervical cancer testing. Since the dependent variable is dichotomous, the decomposition of eq. (8) uses the Erreygers (EI) [59] decomposition method and is expressed as follows:

9 $$EI=4\left[\sum \limits_{j=1}^J{\beta}_j{{\overline{X}}_j}^{\ast }{CI}_j+{GCI}_{\varepsilon}\right]$$

In eq. (9), *β*_*j*_ is the partial effect of each determinant of cervical cancer testing, *CI*_*j*_ is the concentration indices of *X*_*j*_ and *GCI*_*ε*_ is the generalised CI of the error term. The decomposition above works on the assumption of linearity between the variable of interest and its determinants [61] . Weights were used in the data. All the analysis was done in Stata.

## Results

This section presents the results of the paper. Firstly, the descriptive statistics are presented to understand the characteristics of the sample. Table 1 shows that 13% of the samples undertook cervical cancer screening. A minority of the sample was not in any employment (22%). The respondents from urban areas were few (35%), and many of the respondents were married (80%). A majority had primary education (63%). In terms of age, 35% of the respondents were in the category 25–34 years. Almost all the respondents had taken an HIV test at some point in their lives (93%). The southern region had a considerable proportion of the respondents (46%). See Table 1 for more in terms of the distribution of the sample characteristics.

**Table 1:** Social and demographic characteristics

Variable	Variable definition	Mean	Frequency
Cervical cancer screen	1 if had cervical cancer screening, 0 otherwise	13%	1152
Work status	1 if working, 0 otherwise	22%	1975
Location	1 if urban, 0 if rural	35%	3142
Marital status	1 if married, 0 otherwise	80%	7182
No education	1 if no education	13%	1167
Primary education	1 if primary, 0 otherwise	63%	5656
Secondary education	1 if secondary, 0 otherwise	21%	1885
Postsecondary education	1 if postsecondary, 0 otherwise	3%	269
Christian	1 if Christian, 0 otherwise	83%	7452
Muslim	1 if Muslim, 0 otherwise	10%	898
No religion		7%	646
Wealth quintile 1	1 if wealth quintile 1, 0 otherwise	14%	1257
Wealth quintile 2	1 if wealth quintile 2, 0 otherwise	16%	1436
Wealth quintile 3	1 if wealth quintile 3, 0 otherwise	17%	1526
Wealth quintile 4	1 if wealth quintile 4, 0 otherwise	20%	1796
Wealth quintile 5	1 if wealth quintile 5, 0 otherwise	34%	3053
Age group: < 25	1 if age group less than 25, 0 otherwise	24%	2155
Age group: 25–34	1 if age group 25–34, 0 otherwise	35%	3142
Age group: 35–44	1 if age group 35–44, 0 otherwise	22%	1975
Age group: 45–54	1 if age group 45–54, 0 otherwise	12%	1077
Age group: 55+	1 if age group: 55+, 0 otherwise	7%	628
Ever tested for HIV	1 if ever tested for HIV, 0 otherwise	93%	8350
Northern region	1 if from northern region, 0 otherwise	13%	1167
Central region	1 if from central region, 0 otherwise	41%	3681
Southern region	1 if from southern region, 0 otherwise	46%	4130
N		8978	

Moving away from the univariate analysis above, the bivariate analysis is presented in Table 2 to show the distribution of the variables across whether they had undertaken a cervical cancer screening test or not. From Table 2, among those who indicated that they were employed, 16.9% had undertaken a cervical cancer screening test (hence, 83.1% had not) (Chi = 37.42, *P* = 0.00). The urban proportion (21.5%) who had undertaken some cervical cancer test was relatively higher than that of the rural (8.2%). Those with education beyond secondary, 36.7%, indicated that they had undertaken some cervical cancer test. Across wealth quintiles, those in the upper richest quintile (quintile 5) were the largest proportion (23.2%) of those who had cervical cancer screening. Table 2 presents more results on the bivariate relationship.

**Table 2:** Social and demographic characteristics

Variables	Have you ever been tested for cervical cancer?					*P*-value
	No		Yes		Total	
	Frequency	%	Frequency	%	Chi-square	
**Work status**						
Not working	6213	88.3	823	11.7		
Working	1613	83.1	329	16.9	37.42	0.00
**Location**						
Rural	5354	91.8	476	8.2		
Urban	2472	78.5	676	21.5	323.74	0.00
**Marital status**						
Not married	1593	86.8	242	13.2		
Married	6233	87.3	910	12.7	0.26	0.609
**Education status**						
No education	1053	91.3	100	8.7		
Primary	5052	89.3	608	10.7		
Secondary	1559	81.7	350	18.3	222.20	0.00
Post-secondary	162	63.3	94	36.7		
**Religion of the respondent**						
Christian	6428	86.7	989	13.3		
Muslim	823	90.3	88	9.7	10.84	0.00
No religion	575	88.5	75	11.5		
**Wealth quintile**						
Quintile 1	1159	95.3	57	4.7		
Quintile 2	1316	93.9	86	6.1		
Quintile 3	1406	93.1	104	6.9	473.47	0.00
Quintile 4	1602	89	198	11		
Quintile 5	2343	76.8	707	23.2		
**Ever tested for HIV**						
No	619	93.5	43	6.5		
Yes	7207	86.7	1109.00	13.3	25.65	0.00
**Region**						
Northern	999.0	86.3	158	13.7		0.00
Central	3292.0	89.0	406	11.0		
Southern	3535.0	85.7	588	14.3		
Total	7826	87.2	1152	12.8		

Having conducted the above analysis, the proceeding narrative assesses the determinants of cervical cancer screening. Both the Odds ratios (OR) and the 95% confidence interval (95%CI) are are presented in Table 3. Findings shows no significant difference in terms of work status. Furthermore those in urban areas are likely to undertake cervical cancer screening as opposed to those in rural areas (OR = 1.66; 95%CI = (1.36–2.02)). It can also be observed that the respondents who had secondary and post-secondary education were more likely to undertake cervical cancer screening as opposed to those without any education. Apart from the above, being in a higher wealth category was associated with cervical cancer screening compared to the lower wealth group 1. In terms of age, it can be seen that cervical cancer screening was associated with being older than age 24. The results further suggest that those who had ever had an HIV test were more likely to take a cervical cancer screening test (OR 2.48; 95%CI = (1.71–3.60)).

**Table 3:** Determinants of cervical cancer screening uptake in Malawi

Variables	(1)	(2)	(3)	(4)
	Odds ratio	95%CI	Odds ratio	95%CI
Have you ever been tested for cervical cancer?				
Work status	1.13	(0.93–1.36)	1.15	(0.96–1.39)
Location	1.66***	(1.36–2.02)	1.72***	(1.40–2.11)
Marital Status	0.98	(0.81–1.19)	1.01	(0.83–1.23)
Primary education	1.14	(0.88–1.48)	1.14	(0.87–1.48)
Secondary education	1.41**	(1.03–1.93)	1.39**	(1.01–1.91)
Post-secondary education	2.06***	(1.33–3.21)	2.03***	(1.31–3.16)
Christian	1.09	(0.81–1.48)	1.11	(0.82–1.50)
Muslim	0.79	(0.53–1.16)	0.78	(0.53–1.16)
Wealth quintile 2	1.27	(0.87–1.85)	1.24	(0.85–1.81)
Wealth quintile 3	1.31	(0.91–1.88)	1.24	(0.87–1.79)
Wealth quintile 4	1.91***	(1.35–2.69)	1.77***	(1.25–2.50)
Wealth quintile 5	3.43***	(2.39–4.91)	3.14***	(2.19–4.50)
Age group: 25–34	1.87***	(1.44–2.43)	1.90***	(1.46–2.47)
Age group: 35–44	2.83***	(2.16–3.72)	2.87***	(2.18–3.77)
Age group: 45–54	3.14***	(2.31–4.27)	3.16***	(2.32–4.31)
Age group: 55+	2.84***	(1.97–4.11)	2.84***	(1.96–4.10)
Ever tested for HIV	2.48***	(1.71–3.60)	2.42***	(1.67–3.51)
Northern Region			1.17	(0.94–1.46)
Southern Region			0.74***	(0.62–0.88)
Constant	0.01***	(0.00–0.02)	0.01***	(0.01–0.02)
Observations	8978		8978	
Pseudo R2	0.0930		0.0968	
Log Lik	−1.026e+ 06		−1.022e+ 06	
Wald Chi	455.3		455.5	
Pro>Chi2	0.00		0.00	

Robust confidence interval in parentheses.

*** *p* < 0.01, ** *p* < 0.05, * *p* < 0.1

**Fig. 1:**
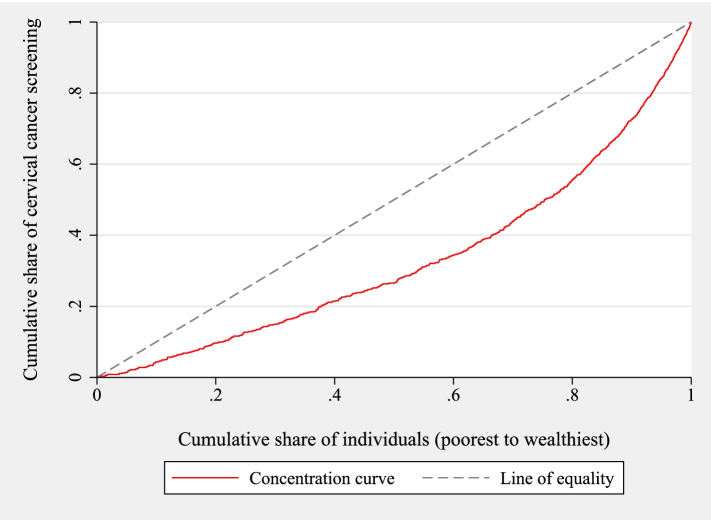
Concentration curve for cancer screening in Malawi

Having presented the determinants, the next step is to present the wealth-related inequality. Figure 1 shows the overall concentration curve for the sample. The concentration curve lies below the line of equality, to the right side. This means that cervical cancer screening was concentrated among individuals from wealthier households.

**Fig. 2:**
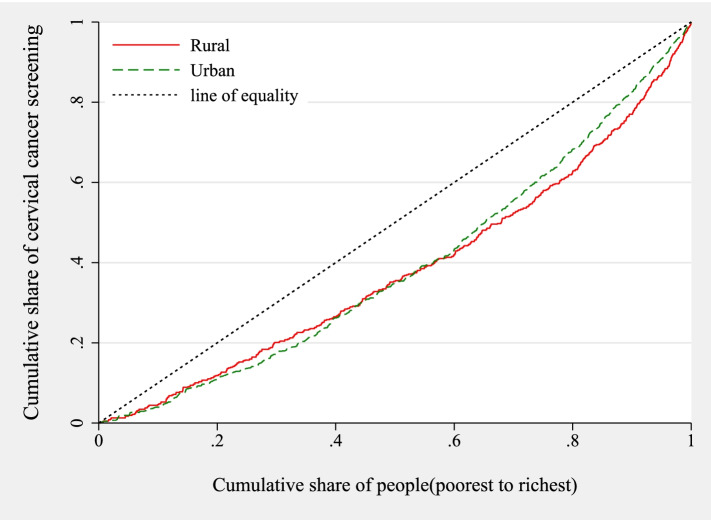
Concentration curves for cervical cancer screening uptake in rural and urban areas in Malawi

Inequality in cervical cancer service uptake was also assessed across rural and urban areas. The results are shown in Fig. 2.

Figure 2 shows the concentration curves for the inequality in cervical cancer testing in rural and urban Malawi. The results from Fig. 2 complement the results in Fig. 1. All the concentration curves are to the right of the line of equality. This suggests that inequality in cervical cancer testing in Malawi exists, and it favours the poor.

**Fig. 3:**
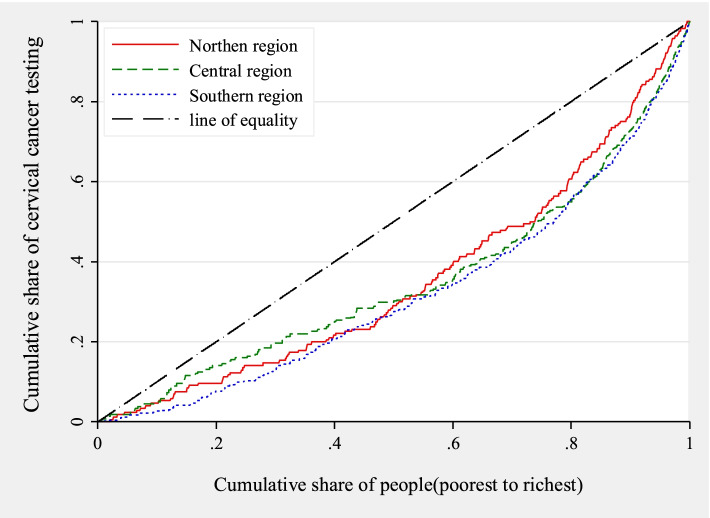
Concentration curves for cervical cancer screening uptake across regions in Malawi

Just as the graph for the rural and urban dimension, socioeconomic inequality in cervical cancer screening is not pro-poor; in other words, since all the concentration curves lie to the right-hand side of the line of equality, it implies higher cervical cancer screening among the rich. See Fig. 3 for more.

Since Figs. 1, 2 and 3 do not indicate the magnitude of inequality and also that the respective graphs intersect at some point; therefore, it is important to have a summary measure. As such, the EI was calculated for each of the respective three regions as well as rural and urban locations. The results are presented in Table 4.

**Table 4:** Concentration indices for cervical screening in various regions, rural and urban locations

	Overall	95% CI	Rural	95% CI	Urban	95% CI
Concentration Indices	0.142***	[0.127,0.156]	0.075***	[0.059,0.090]	0.195***	[0.162,0.228]
*N*	8943		5806		3137	
	**Northern region**	95% CI	**Central** region	95% CI	**Southern** region	95% CI
Concentration Indices	0.168***	[0.122,0.215]	0.100***	[0.080,0.120]	0.160***	[0.138,0.181]
*N*	1155		3686		4102	

95% confidence intervals in brackets, * *p* < 0.10, ** *p* < 0.05, *** *p* < 0.01.

As can be seen from Table 4, the overall EI without data disaggregation was 0.142 (95%CI: 0.127–0.156) and was significant at a 1% level. This means that cervical cancer screening uptake is concentrated among the rich. In terms of the rural and urban divide, the rural EI was 0.075 (95%CI 0.059–0.090; *p* < 0.01), whereas the urban EI was 0.195 (95%CI; 0.162–0.228; *p* < 0.01). The magnitude for the rural EI suggests more inequality in urban areas than in rural areas. Regarding regional variation (North, Central, South), all the EI were positive and significantly different from zero at the 1% level.

In order to understand how the observed inequality in the aforementioned socioeconomic factors contributes to the inequality in the uptake of cervical cancer screening, a decomposition of the overall concentration index was undertaken. Table 5 shows the results from the decomposition of the EI for cervical cancer screening uptake.

**Table 5:** Decomposition of the concentration index for cervical cancer screening

Variable	1	2	3	4
	CI	Elasticity	Contribution	Percentage
Working	0.0698	0.0197	0.0007	0.50
Location	0.6627	0.1664	0.0566	40.14
Marital status	0.0332	0.0552	0.0009	0.67
Primary education	−0.0687	0.1091	−0.0038	−2.73
Secondary education	0.4305	0.0821	0.0181	12.87
Postsecondary education	0.8443	0.0242	0.0105	7.44
Christian	0.019	0.0884	0.0009	0.61
Muslim	−0.0778	−0.0147	0.0006	0.42
Age group: 25–34	0.0459	0.1177	0.0028	1.97
Age group: 35–44	0.0237	0.1554	0.0019	1.34
Age group: 45–54	0.0217	0.1022	0.0011	0.81
Age group: 55+	−0.0198	0.0552	−0.0006	−0.40
Ever tested for HIV	0.0047	0.4279	0.001	0.73
Northern region	0.194	0.0248	0.0025	1.75
Southern region	−0.074	−0.0978	0.0037	2.64
	**A**	Explained socioeconomic inequality **(A)**	0.097	
	**B**	Total socioeconomic inequality (B)	0.141	
	**C**	Residual (**B-A)**	0.044	

As observed from Table 5, the total inequality was about 0.140, of which the individual characteristics explain almost 0.097 (see under column 3, row C). Much of the contribution of inequality in cervical cancer screening uptake emanates from location (living in urban area), accounting for 40.14%. The education levels also account for an aggregate of 17.58%. This means the residual (0.044), implying that the model itself explains a large proportion of the inequality in cervical cancer screening uptake. See Table 5 for more analysis on how the respective socioeconomic factors contribute to the inequality in cervical cancer screening uptake.

## Discussion

This paper represents the first empirical evidence of socioeconomic inequality in cervical cancer screening uptake in Malawi. The paper draws on the data from the nationally representative MPHIA conducted in 2015 and 2016. This is advantageous because it also undertakes the study four years after the Malawi government scaled up cervical cancer screening services in the country. Given the current debate surrounding the burden of cervical cancer, exploring the socioeconomic inequality in cervical cancer screening is particularly relevant to Malawi as well as internationally –including the donor community who have been at the helm of programme support for cervical cancer. The salient findings on determinant analysis and the decomposition analysis are presented in the subsequent paragraphs.

On determinant analysis, this study does not identify a statistically significant association between religion, employment and the uptake of cervical cancer screening. It is interesting to note that despite the scale up of cervical cancer screening, geographical location also presents itself as a hindrance to the uptake of cervical cancer screening, as was the case in Botswana [62]. In this respect, the findings show that women in rural areas are less likely to undertake cervical cancer screening than women in urban areas, which corroborates what was found in Kenya [63]. Furthermore, the finding of this paper is in accordance with some findings which were established in terms of cervical cancer screening uptake among women living with HIV in Malawi [19, 64].

Moreover, this finding supports the established results in many high-income countries in, for example, Europe and America, where factors that have been seen to drive these factors include lack of regular medical follow-up, costs associated with pap smears and age, [65–68], to mention a few. Furthermore, supply-side heterogeneities in the distribution of health personnel have also contributed to the problem. The geographical heterogeneity in health practitioners may be akin to the problem of lack of personnel in some of the facilities in Malawi. Thus, our results suggest the existence of “Inverse Care Law” in the use of cervical cancer screening services. However, it is important to state that the institutional differences between the developed, high-income countries and the low-income countries may render the comparison at some point, and should be undertaken cautiously. The causative factor for this still remains speculative. It could be an issue with lack of awareness of what is offered in the health facilities in rural areas or that many consider it an obstacle to travel to distant areas to seek medical care [69].

Still, the determinants age and wealth status are positively associated with cervical cancer screening uptake. Regarding age, it is common that most young women consider cervical cancer a problem for the elderly. As a result, many young women are not interested in taking a screening test. With respect to wealth status, an explanation for this result may be similar to what was found in Uganda [70]. In the Uganda study, it was argued that those women who can afford transport/food are likely to undertake cervical cancer screening. Furthermore, it is important to note that in Malawi, the economic situation is exacerbated by the fact that a large proportion (50%) of Malawians live under the poverty line and on less than 2 USD per day (NSO, 2017b) and that a recent rise in inequality in education, health and economic advantage has put poor people at a considerable disadvantage [71].

As indicated before, apart from the determinant analysis, socioeconomic inequality analysis and decomposition analysis indicated some informative findings. The results show that the concentration index for socioeconomic inequality in cervical cancer screening uptake is positive, which means less uptake in cervical cancer screening among poor members of the Malawian society. While there is some evidence that suggests that cervical cancer screening appears to be concentrated among the wealthy [35], the magnitude of the inequality is still not emphasised. The decomposition analysis also revealed a few factors that explain the large share of the socioeconomic inequalities in cervical cancer screening. These included education from secondary school and above, and living in an urban area (which explained the largest over 17.58%). Although there is no direct comparable study on decomposition of inequality in cervical cancer screening uptake for Malawi, the findings from the analysis can be linked to certain studies, which may help to understand why such is the case.

It can be speculated that the lack of awareness among many rural women regarding cervical cancer screening could potentially be a factor. Furthermore, most women living in deprived households with low levels of schooling and with a disproportionately lower level of knowledge experienced the lowest utilisation of screening services for cancer [35, 36, 72]. Health system challenges may also be large contributors to the locational factor, which contributes to the observed inequality. This has also been noted as one factor contributing to the low uptake of many services, including cervical cancer screening. There are about 500 health facilities in Malawi. Implementing cervical cancer screening in a population where almost 52% are women is a challenge with such a relatively small number of facilities. Occasionally, there is even a shortage of essential supplies needed for cervical cancer, such as vinegar, and space, among other things [64].

Although this paper has produced interesting results, it is worth mentioning the current study’s limitations. First, the current methods do not consider the influence of unmeasured variables. This is because the data did not correct them. Secondly, the interrelationships between the variables were not considered because the concentration indices methods are not designed to deal with such. Therefore, decomposition methods are not considered to be casual and, thus, the results are not necessarily causal, given that the methods used are not designed to address causality. The issues of interrelations and unmeasured variables may raise the problem of endogeneity [73]. The ideal solution would be to use methods such as instrumental variables [74], but no plausible instrument was available in the current data. The difference-in-difference [75] method would have also been appropriate, but the challenge was that only data for one wave were available. Furthermore, apart from the mentioned limitations, the information on testing is self-reported; as such, there may be a likelihood of reporting bias led by societal expectations (especially for HIV and cervical testing). This problem cannot be addressed by the methods used in this paper.

## Conclusion

This paper has established that cervical cancer screening is concentrated among wealthy individuals, despite being free at the point of use in public facilities. Socioeconomic inequalities in the uptake of cervical cancer screening is prevalent in Malawi to the advantage of wealthy women. These inequalities are driven mainly by crucial social determinants of health inequalities such as education, wealth and place of residence. To cater for policy and using this paper as a source of vital information, there is a need to include studies on cervical cancer screening in the secondary-school curriculum. It would also be appropriate to have special sensitisation days for cervical cancer, especially in schools, so that pupils learn about the subject beyond the classroom.

## Supplementary Information

**Additional file 1.**

## Data Availability

The datasets generated and/or analysed during the current study are publicly available at https://phia-data.icap.columbia.edu/, and free to download.
