# Supplementary material for: Explaining socioeconomic inequality in cervical cancer screening uptake in Malawi
Source: BMC Public Health. 2022 Jul 18;22:1376. doi: 10.1186/s12889-022-13750-4 (PMC9295286; doi:10.1186/s12889-022-13750-4)
Supplement: Supplementary file 1 — Additional file 1. [file 12889_2022_13750_MOESM1_ESM.docx]

## **Appendix 1 - Wealth index variables used in the data**

The variables used as indicators for wealth in the MPHIA data were:

| **Indicator variable** | **Type** | **Description** |
| --- | --- | --- |
| memsleep | Numeric (count) | Number of household members per sleeping room |
| MatRoof | Categorical | Dwelling roofing material |
| MatExWalls | Categorical | Dwelling wall material |
| matfloor | Categorical | Dwelling floor material |
| ToiletType | Categorical | Type of toilet used by the household |
| ToiletShare | Binary | Is the toilet shared with other households? |
| watersource | Categorical | Source of water used by the household |
| CookingFuel | Categorical | Type of cooking fuel used by the household |
| *For the remaining variables:* | | *Does this household have/own…?* |
| HAVEBEDMAT | Binary | A bed with a mattress |
| HAVEELECT | Binary | Electricity connected |
| HAVELAMP | Binary | A paraffin lamp |
| HAVEPHONE | Binary | A non-mobile telephone |
| HAVERADIO | Binary | A radio in working condition |
| HAVEREFRIG | Binary | A refrigerator in working condition |
| HAVESOFA | Binary | A sofa set |
| HAVETABLE | Binary | A table and chair |
| HAVETELE | Binary | A television in working condition |
| OWNBIKE | Binary | A bicycle |
| OWNCAR | Binary | A car |
| OWNCHIKN | Binary | Chickens or other poultry |
| OWNCOW | Binary | Cattle |
| OWNGOAT | Binary | Goats |
| OWNMOTO | Binary | A motorcycle or motor scooter |
| OWNPIG | Binary | Pigs |
| OWNSHEEP | Binary | Sheep |
| OWNWATCH | Binary | A watch |
